# Lack of selective resistance of influenza A virus in presence of host-targeted antiviral, UV-4B

**DOI:** 10.1038/s41598-019-43030-y

**Published:** 2019-05-16

**Authors:** Kelly L. Warfield, Kaitlyn R. Schaaf, Lisa Evans DeWald, Kevin B. Spurgers, Wei Wang, Eric Stavale, Michelle Mendenhall, Meghan H. Shilts, Timothy B. Stockwell, Dale L. Barnard, Urban Ramstedt, Suman R. Das

**Affiliations:** 1 Emergent BioSolutions, Gaithersburg, MD 20879 USA; 2 Department of Infectious Diseases, Vanderbilt University Medical Center, Nashville, TN 37232 USA; 3 Infectious Diseases Group, J. Craig Venter Institute, Rockville, MD 20852 USA; 4 Integrated Biotherapeutics Inc., Gaithersburg, MD 20878 USA; 5 Institute for Antiviral Research, Utah State University, Logan, UT 84322-5600 USA; 6 Unither Virology, LLC, Silver Spring, MD 20910 USA; 7 Present Address: Cellular Biology and Viral Immunology Section, DIR, National Institute of Health, Bethesda, MD 20892 USA; 8 Present Address: Abviro, 4800 Hampden Lane, Bethesda, MD 20814 USA; 9 Present Address: National Biodefense Analysis and Countermeasures Center, 8300 Research Plaza, Fort Detrick, MD 21702 USA

**Keywords:** Antiviral agents, Influenza virus

## Abstract

Development of antiviral drug resistance is a continuous concern for viruses with high mutation rates such as influenza. The use of antiviral drugs targeting host proteins required for viral replication is less likely to result in the selection of resistant viruses than treating with direct-acting antivirals. The iminosugar UV-4B is a host-targeted glucomimetic that inhibits endoplasmic reticulum α-glucosidase I and II enzymes resulting in improper glycosylation and misfolding of viral glycoproteins. UV-4B has broad-spectrum antiviral activity against diverse viruses including dengue and influenza. To examine the ability of influenza virus to develop resistance against UV-4B, mouse-adapted influenza virus was passaged in mice in the presence or absence of UV-4B and virus isolated from lungs was used to infect the next cohort of mice, for five successive passages. Deep sequencing was performed to identify changes in the viral genome during passaging in the presence or absence of UV-4B. Relatively few minor variants were identified within each virus and the ratio of nonsynonymous to synonymous (dN/dS) substitutions of minor variants confirmed no apparent positive selection following sustained exposure to UV-4B. Three substitutions (one synonymous in PB2, one nonsynonymous in M and PA each) were specifically enriched (>3%) in UV-4B-treated groups at passage five. Recombinant viruses containing each individual or combinations of these nonsynonymous mutations remained sensitive to UV-4B treatment in mice. Overall, these data provide evidence that there is a high genetic barrier to the generation and selection of escape mutants following exposure to host-targeted iminosugar antivirals.

## Introduction

Rapidly acquired genetic variability due to error-prone polymerases has made rational drug development against RNA viruses, such as influenza virus, arduous. The fundamental problem underlying this difficulty is the inevitable development of drug resistance, where changes in a very small number of amino acid residues in the targeted viral protein is sufficient to reduce or completely block efficacy of a drug. For example, a single amino acid substitution (H274Y) in influenza A virus (IAV) isolates confers resistance to the neuraminidase inhibitor, oseltamivir^1^. Rapid development of resistance necessitates new approaches to the development of antiviral drugs. Since viruses are obligate intracellular parasites, they are critically dependent upon host factors for infection, replication, and spread. Identification and targeting of host factors that are critical for the viral replication cycle provides an opportunity for the development of novel classes of antiviral drugs^2,3^. Due to the improbability of changes to the host genome during an acute viral infection cycle, evasion of host-directed antiviral drugs is much less likely to occur.

IAV is a good target for the development of a host-directed antiviral therapy as the host molecular pathways that interact with the immunodominant viral proteins, hemagglutinin (HA) and neuraminidase (NA), have been well described. HA and NA are the two major surface glycoproteins of IAV that play essential roles in virion attachment and budding^4,5^. HA attaches the incoming virions to target cells by binding terminal sialic acid residues on cell-surface glycans^5^, whereas NA is a sialidase that assists with the budding process by cleaving sialic acid on the cell surface^6^. As a prototypical homotrimeric type I integral membrane protein, HA is synthesized in the endoplasmic reticulum (ER) of infected cells and transported through the Golgi complex to the plasma membrane, where it is incorporated into budding virions^7–9^. A variable number (dependent on the strain) of N-linked oligosaccharides are added co-translationally to HA as it is extruded into the ER through the translocon, and are subsequently trimmed and modified extensively during transport to the cell surface^8,10–12^. HA folding begins co-translationally, as demonstrated by the acquisition of intrachain disulfide bonds and the binding of monoclonal antibodies (mAbs) specific for discontinuous epitopes within HA to nascent chains^8,11–15^. N-terminal glycosylation at the globular head of HA helps the virus escape the immune response, while glycosylation of some sites at the stem of HA are critical for protein folding and stability^11,13–15^. Similarly, NA is a transmembrane tetrameric protein that uses the ER-Golgi route to the cell surface and is glycosylated in the process^16^. As both HA and NA rely heavily on glycosylation and other processing in the ER, a host-directed drug that inhibits this process has high potential for antiviral activity.

Key targets for inhibiting the host ER glycosylation pathway include the α-glucosidase I and II enzymes. These enzymes are responsible for making modifications to the co-translationally attached N-linked oligosaccharides, which are necessary for the proper folding of many glycoproteins^17,18^. Enveloped viruses that express surface glycoproteins, such as HA and NA of IAV, are dependent on the host cell α-glucosidase I and II enzymes for their replication. If viral proteins are not properly glycosylated, protein folding, stability, functionality, and immune evasion are impaired, and may result in reduced viral secretion or the production of defective virions^8–11^.

Iminosugars are glucomimetics with structural similarity to sugar molecules that can competitively inhibit glucosidase enzymes. Some iminosugars specifically target the α-glucosidases in the ER^19^. As a result, their therapeutic potential has been investigated against a range of viruses both *in vitro* and *in vivo*^20–23^. The iminosugar UV-4B has demonstrated *in vitro* and *in vivo* activity against a phylogenetically diverse set of glycosylated, enveloped viruses, including dengue (DENV) and influenza viruses^24–28^. It was previously demonstrated that DENV has a high genetic barrier for development of resistance against UV-4B^27^.

Here, we assessed the development of viral resistance to UV-4B treatment *in vivo* using a murine model of IAV infection. Mouse-adapted influenza A/Texas/36/91 (H1N1) was passaged in mice treated with UV-4B or vehicle for five successive passages. The sensitivity of the 5-times passaged viruses (P5) to treatment with UV-4B or the unrelated antiviral oseltamivir, which is currently approved for use to treat IAV infections, was confirmed in mice. The passaged viruses were deep-sequenced and relatively few minor variants were identified within each virus. Three substitutions (one synonymous in PB2 and two nonsynonymous in M and PA) were specifically enriched in P5 viruses passaged in the presence of UV-4B. However, these substitutions did not impact the efficacy of UV-4B or oseltamivir against the P5 viruses as evident from *in vivo* efficacy studies. Recombinant viruses containing each individual or combinations of these mutations remained susceptible to UV-4B treatment, showing no increased replication *in vitro* or enhanced disease severity *in vivo*.

## Results

### Passaging IAV *in vivo* in the presence of the host-targeted antiviral UV-4B does not decrease susceptibility to the drug

**Figure 1:**
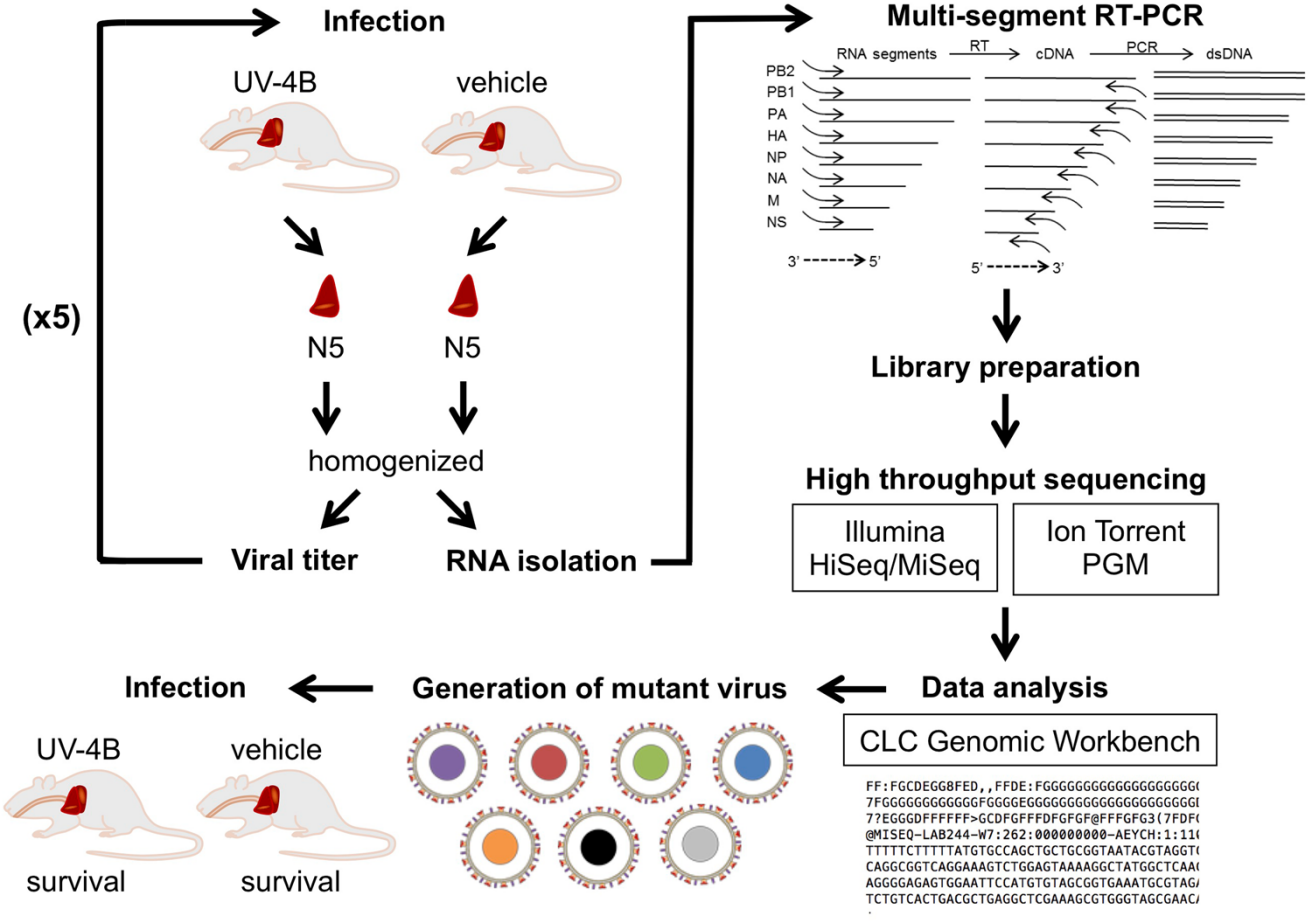
Schematic diagram of the study design. Two groups of 15 female BALB/c mice were challenged i.n. with ~1 LD_90_ (~52 PFU) of mouse adapted A/Texas/36/91 (H1N1) and treated by intragastric administration with 100 mg/kg UV-4B or vehicle (water) TID for 7 days, starting 1 h after infection. Mice from each group (n = 5) were sacrificed on day 4 post-infection and their lungs were isolated and homogenized. A portion of the lung homogenates were pooled by group and used as the challenge virus (~1 LD_90_ or ~52 PFU/mouse) for the next passage in mice, successively for a total of 5 passages. The remaining portion of the lung homogenates were used to measure viral titer and isolate RNA for amplification by multi-segment RT-PCR. Sequencing libraries were prepared and sequenced on either the Illumina HiSeq 2000 or Illumina MiSeq v2 instruments (with repeat sequencing on the Ion Torrent PMG). Virus sequence assembly and identification of SNPs were performed using the CLC Genomics Workbench. Mutant viruses recapitulating the corresponding nucleotide changes of 3 SNPs identified (individually and in combination) were generated using site directed mutagenesis. Lethality and susceptibility to UV-4B of the mutant viruses was measured *in vivo* using similar experimental conditions.

**Figure 2:**
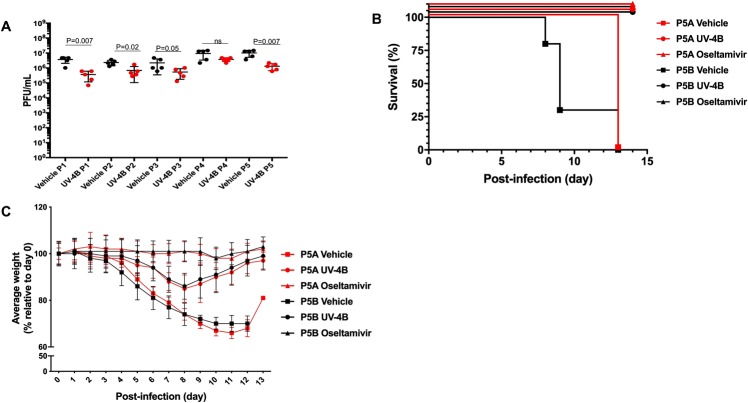
Properties of IAV after *in vivo* passaging in the presence or absence of UV-4B. (**A**) Mouse-adapted INFV A/Texas/36/91 (H1N1) was passaged five times in the presence or absence of UV-4B. BALB/c mice were infected i.n. with the P0 parent virus or virus isolated from lungs of infected mice after 1 (P1) to 4 (P4) passages and treated by intragastric administration with 100 mg/kg UV-4B or vehicle (water) TID beginning 1 h after infection. Lungs were isolated from mice (n = 5/group) on day 4 post-infection and viral titers were measured by plaque assay after each passage in mice. UV-4B-treated groups in passages (P)0–4 were significantly lower (p < 0.05) than vehicle-treated groups at each passage, except for P4 (p = 0.052). (**B**,**C**) Survival outcome and relative weight of mice infected with IAV passaged 5 times *in vivo* in the presence or absence of UV-4B. BALB/c mice (n = 10/group) were exposed i.n. to virus (~1 LD_90_) that was passaged 5 times in mice in the presence of UV-4B (P5A) or water (P5B), and treated via intragastric administration with UV-4B (100 mg/kg, TID), water (TID), or oseltamivir (20 mg/kg, twice daily) beginning 1 h after challenge. Survival (**B**) and relative average weight compared to day 0 (**C**) were measured daily through the end of the study (Day 14).

A murine model of IAV infection was used to test for the development of viral resistance to the iminosugar UV-4B. The dosing route and regimen were selected based on available data from tolerability and pharmacokinetic studies in uninfected mice^25,28^ and previous efficacy studies using IAV murine models of disease^24,25^. Groups of mice were challenged intranasally (i.n.) with influenza A/Texas/36/91 (H1N1) (passage 0; P0) and treated by intragastric administration of UV-4B or vehicle three times a day (TID) for seven days (Fig. 1). A portion of mice (ten) in each group were observed for morbidity and mortality for 14 days. The remaining five mice in each group were sacrificed on Day 4 post-infection (p.i.) and their lungs were harvested and homogenized for virus titration and deep sequencing. Following virus titration of the lung homogenates, a portion of the homogenates were pooled by group and used as the challenge virus for the next passage (P1) in mice. Virus passaging continued for a total of five successive passages. As expected, and similar to our previously published work^24,25^, viral titers in the lungs of UV-4B-treated mice were significantly lower (P ≤ 0.05) than that of vehicle-treated mice after each passage except for passage 4 (P = 0.052) (Fig. 2A).

An efficacy study was performed to determine whether IAV isolated after 5 passages (P5) from animals treated with UV-4B (P5A) or vehicle (P5B) were still sensitive to treatment with UV-4B or the influenza virus (INFV) antiviral oseltamivir. Mice were challenged i.n. with P5A or P5B IAV and then treated with UV-4B (100 mg/kg, TID), vehicle (water), or oseltamivir (20 mg/kg, twice daily). The UV-4B treatment dose selected was based on previous mouse efficacy studies^25,26^. Mice were observed daily for changes in health, weight and mortality for 14 days. All mice treated with UV-4B or oseltamivir survived infection with P5A and P5B viruses (Fig. 2B). All mice infected with P5A or P5B IAV and treated with vehicle succumbed to disease with a median survival time of 13 or 9 days, respectively (Supplemental Table 1). There were no differences in the average decrease in body mass of similarly treated groups infected with either P5A or P5B viruses (Fig. 2C). The onset and severity of clinical signs of disease (health score) were comparable between groups receiving similar treatments whether they were infected with the P5A or P5B viruses. Overall, this suggests that IAV passaged in mice five times in the presence of UV-4B did not acquire mutations that could potentially cause resistance to UV-4B or oseltamivir treatment or increase *in vivo* pathogenicity.

### Sequencing of IAV identified limited selective mutations acquired during *in vivo* passaging in the presence of UV-4B

IAVs from the five individual mice at each passage that were treated with UV-4B (n = 25 samples, 5 per passage) or vehicle (n = 25 samples, 5 per passage), along with the input challenge virus, were deep-sequenced to achieve at least 200-fold coverage at each base of the coding sequence. Variant analysis of deep-sequence data was performed to identify selection of variants in UV-4B-treated animals versus vehicle-treated animals. The consensus sequence for the parental input mouse-adapted influenza A/Texas/36/91 (H1N1) challenge virus used for this study was previously unpublished. Therefore, we compared it against 2 deposited consensus sequences in the National Center for Biotechnology Information (NCBI) database for the A/Texas/36/91 (H1N1) strain. Five nonsynonymous nucleotide differences were found in the HA and NA sequences for the input parental virus compared with both NCBI sequences, only one of which (HA residue N104D) removes a potential N-linked glycosylation sequon (Supplemental Table 2).

Deep sequencing analysis of the *in vivo*-passaged IAV was performed to determine the consensus sequence and analyze consensus differences and low frequency nucleotide variations (quasi species/ minor variants) observed in the IAV samples to identify potential genomic population variation in UV-4B-treated and vehicle-treated mice. The sequence analysis identified that the genomic population in the virus samples is fairly uniform for an RNA virus (relatively few minor variants) and most were not specifically associated with UV-4B treatment. There were 31 selectively enriched coding sequence variants with >3% Single Nucleotide Polymorphisms (SNPs) identified at P5 which were unequally divided among 7 of the 8 RNA segments (Table 1). A cut off of >3% was considered true polymorphism (with minimum of 200X coverage at each base) to account for errors we observed from systematic sequencing (as high as 1%) with the Illumina platform, in addition to errors due to reverse transcription and library preparation^29,30^.

**Table 1:** Summary of selectively enriched coding sequence variants at passage five.

RNA Segment (nucleotide)	Gene Product (no of amino acids)	Synonymous Substitutions	Non-synonymous Substitutions	Associated with UV-4B Treatment
1 (2341)	Polymerase PB2 (759)	4	3	1 (S)
2 (2341)	Polymerase PB1 (757)	1	1	1 (S)
3 (2233)	Polymerase PA (716)	4	5	3 (NS)
4 (1778)	Hemagglutinin HA (566)	1	2	0
5 (1565)	Nucleoprotein NP (498)	1	2	1 (NS)
6 (1413)	Neuraminidase NA (454)	2	0	0
7 (1027)	Matrix M1 (252) M2 (97)	1	4	2 (NS)
8 (890)	Non-structural proteins NS1 (230) NS2 (121)	N/R	N/R	N/A

S = synonymous, NS = nonsynonymous, N/R = none reported, N/A = not applicable.

The summary of results for P5 sequence changes are shown in Table 2. Fourteen SNPs did not result in an amino acid substitution (i.e., synonymous). Of the 17 nonsynonymous substitutions, 6 were enriched in samples from UV-4B-treated mice. The six nonsynonymous substitutions associated with UV-4B treatment were limited to the polymerase acidic (PA), nucleoprotein (NP), and matrix (M) proteins. Other investigators have observed mutations in PA, polymerase basic 2 (PB2), and NP, which were attributed to mouse adaptation; however, not specifically at these amino acid positions identified here^31^. Only two of the six nonsynonymous substitutions (one each in M and PA) and one synonymous substitution in PB2 were specific to UV-4B treatment and were not also present in viruses from the vehicle-treated samples.

**Table 2:** Selectively enriched coding sequence variants of influenza A/Texas/36/91(H1N1) virus in untreated vs. UV-4B-treated P5 mice.

RNA Segment	Gene	NT position	AA position	Passage 5 (P5) mice		
				Untreated counts major/minor	UV-4B-treated counts major/minor	Functional impact^a^
1	PB2		237		2 A/G, 3 G/A^c^	subst_synonymous[GGA:G GGg:G c]
2	PB1	93	27	2 C/T	5 C/T^d^	subst_synonymous[GAC:D GAt:D c]
3	PA		626		3 A/G, 2 G/Ac	subst_NONSYNONYMOUS[AAA:K AgA:R c]
4	PA	542	177	1 C/T	4 C/T, 1 T/C^d^	subst_NONSYNONYMOUS[ACC:T AtC:I c]
4	PA-X	542	177	1 C/T	4 C/T, 1 T/C^d^	subst_NONSYNONYMOUS[ACC:T AtC:I c]
4	HA	126	36	5 G/T^b^	2 G/T	subst_NONSYNONYMOUS[GTA:V tTA:L c]
4	HA	1160	380	5 G/A^b^	2 G/A	subst_synonymous[GCG:A GCa:A c]
5	NP	133	34	3 G/A	3 A/G, 2 G/A^d^	subst_NONSYNONYMOUS[GGT:G aGT:S c]
6	NA	1115	369	5 G/A^b^	4 G/A	subst_synonymous[AAG:K AAa:K c]
7	M1	757	248	2 G/T	5 G/A^c^	subst_NONSYNONYMOUS[ATG:M ATa:I c]
7	M2	757	19	2 G/T	5 G/A^c^	subst_NONSYNONYMOUS[TGC:C TaC:Y U]

AA – Amino Acid; NT – Nucleotide.

^a^The bracketed annotation refers to the majorCodon:majorResidue minorCodon:minorResidue conserved(c)/UNCONSERVED(U) amino acid substitution.

^b^Indicates selective enrichment of minor variants in untreated mice that are also present in the UV-4B-treated group, but in fewer mice.

^c^Indicates selective enrichment of minor variants in UV-4B-treated mice and no equivalent changes noted in the untreated group.

^d^Indicates that all the passage 5 UV-4B-treated mice had the minor variant, which in some cases became the major variant, but less than five untreated mice had the same minor variant.

**Figure 3:**
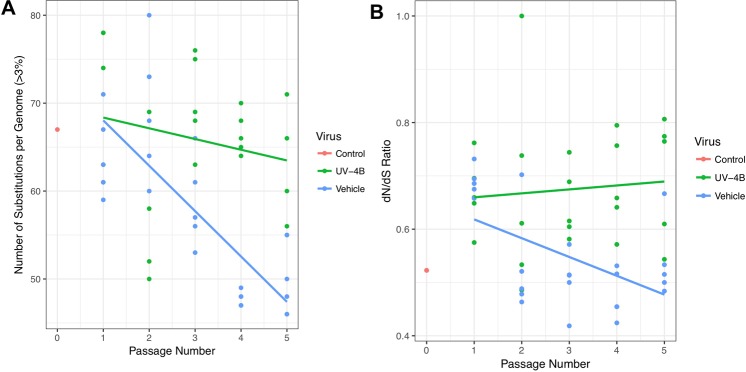
Regression lines plotting the relationship between passage number and either (**A**) number of substitutions per genome (>3%) or (**B**) the dN/dS ratio (number of nonsynonymous substitutions divided by the number of synonymous substitutions). Analysis was performed using the R package ggplot2 with method “lm” (linear model).

Plotting of the substitution rate (minor variant >3%) for each passage shows no significant change in the rate of substitution over passaging in UV-4B-treated mice (linear regression analysis of non-zero slope p-value = 0.2795). In contrast, linear regression analysis shows a significant decrease in the number of substitutions in vehicle-treated mice (p-value < 0.0001) (Fig. 3A). Unlike what has been observed with direct antivirals (e.g., oseltamivir)^32^, we found neutral selection in viruses from UV-4B treated mice based on the ratio of nonsynonymous to synonymous substitutions (dN/dS), despite the constant number of substitutions over time (linear regression of non-zero slope p-value = 0.65) (Fig. 3B), and negative selection was observed in vehicle-treated mice over successive passages (linear regression of non-zero slope p-value = 0.0063). Regardless of being under pressure, as evident from neutral (UV-4B group) *vs*. negative (vehicle group) selection, the lack of any positive selection in the presence of UV-4B after 5-pasages further confirms the high genetic barrier for the emergence of drug resistance in the presence of host-targeted antivirals (e.g. UV-4B)

### Recombinant IAV with substitutions enriched in P5 show no increased replication in MDCK cells

**Figure 4:**
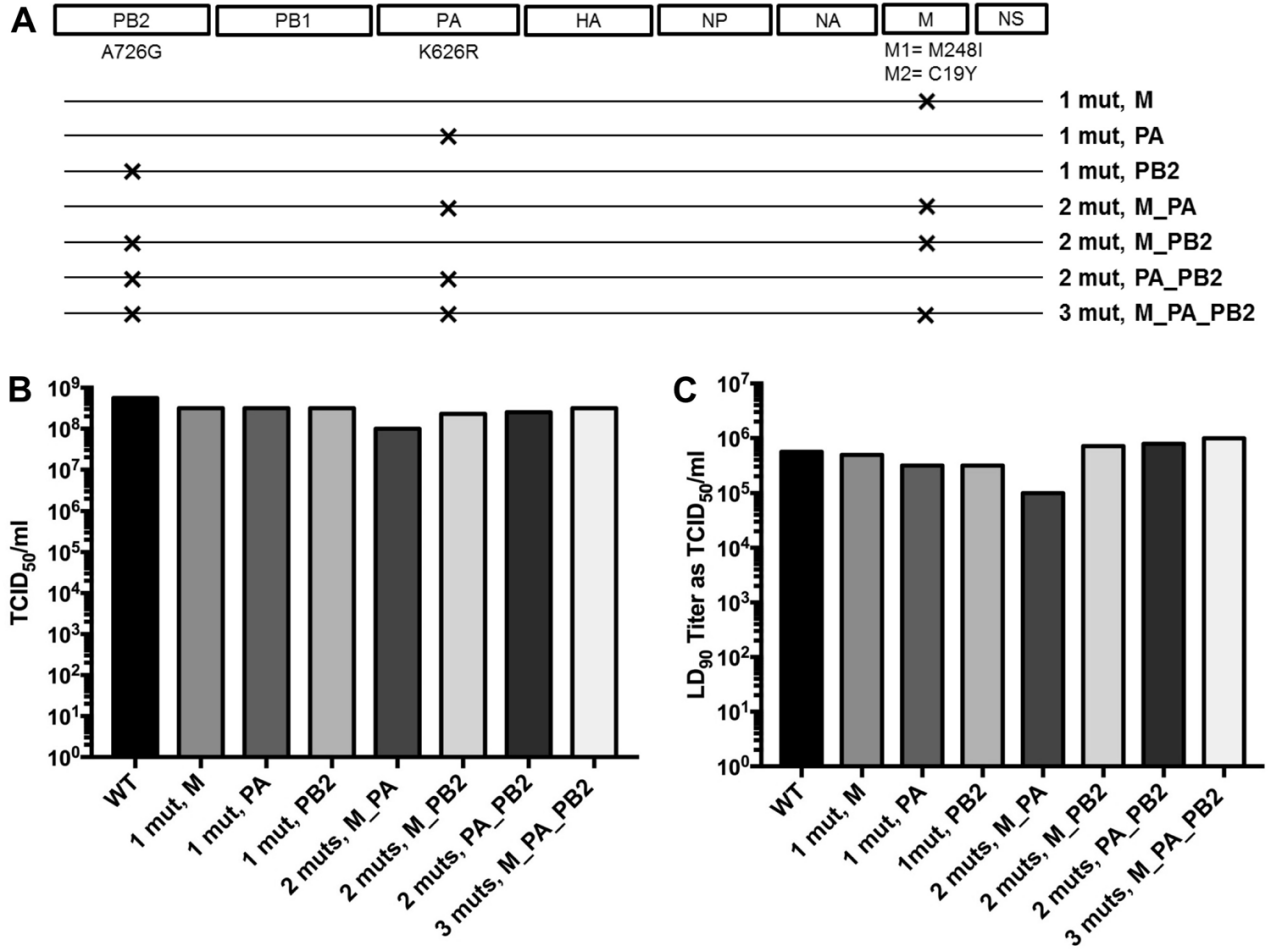
*In vitro* and *in vivo* replication efficiency of recombinant wild-type and mutant viruses. (**A**) Schematic of recombinant viruses generated. (**B**) Viral titers for wild-type and recombinant mutant viruses were determined by TCID_50_ assay in MDCK cells at Day 4. (**C**) Mice were infected i.n. with decreasing challenge doses of wild-type or mutant viruses. Survival was monitored to the end of the study (Day 14) and the LD_90_ was determined for each virus.

All 8 gene segments of A/Texas/36/91 (H1N1) virus were synthesized and cloned to develop reverse genetics systems for further evaluation^33^. M, PA, and PB2 gene segments were mutagenized to generate the three specifically enriched substitutions found in P5A viruses to determine whether these acquired substitutions have any significant selective advantage for the virus to escape UV-4B treatment. The nonsynonymous substitution in the M gene segment results in an amino acid substitution in both M1 (M248I) and M2 (C19Y), whereas the nonsynonymous substitution in PA results in the amino acid change K626R. We also generated a synonymous nucleotide substitution in PB2 (A726G). A total of 8 recombinant (r) mouse-adapted influenza A/Texas/36/91 (H1N1) viruses were rescued (Fig. 4A) using an established reverse genetics rescue system^34,35^.

### No selective advantage of the substitutions enriched in P5 over the input mouse-adapted wild-type virus

Titration of viruses in Madin-Darby Canine Kidney (MDCK) cells after rescue showed very similar titers for each of the recombinant mutant viruses, demonstrating that these substitutions were not detrimental to viral replication/fitness *in vitro* (Fig. 4B). Next, we compared the *in vivo* lethality of recombinant IAVs containing substitutions that were enriched in the P5A virus to that of recombinant wild-type mouse-adapted influenza A/Texas/36/91 (H1N1) virus in BALB/c mice. Mice were infected i.n. with decreasing doses of recombinant viruses, and changes in weight loss and survival were monitored. Less pronounced weight loss and a higher survival rate were observed with decreasing challenge doses in a largely dose-dependent manner (Supplemental Figs 1 and 2). Overall, the LD_90_ titers (converted as TCID_50_ titer) were similar among wild-type and mutant viruses in BALB/c mice (Fig. 4C and Table 3), suggesting that no increase in lethality was acquired by viruses with these specific mutations.

**Table 3:** Estimated 90% lethal dose (LD_90_) of various influenza A (H1N1) viruses in BALB/c mice treated with water three times daily for 7 days.

Virus ID	LD_90_ Dilution Factor	LD_90_ (as TCID_50_ Titer)
rA/Texas/36/91 (WT)	1:1000	562341
rA/Texas/36/91(1 mut M)	1:640	494105
rA/Texas/36/91 (1 mut PA)	1:1000	316227
rA/Texas/36/91 (1 mut PB2)	1:1000	316227
rA/Texas/36/91 (3 muts M_PA_PB2)	1:320	988211
rA/Texas/36/91 (2 muts M_PA)	1:1000	100000
rA/Texas/36/91 (2 muts M_PB2)	1:320	715896
rA/Texas/36/91 (2 muts PA_PB2)	1:320	784964

### Substitutions enriched in P5A are sensitive to both UV-4B and oseltamivir

**Figure 5:**
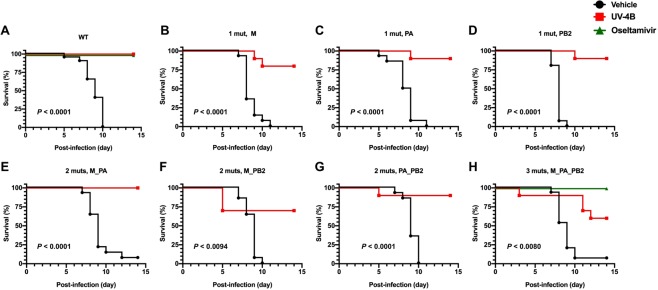
Survival outcome of mice infected with recombinant influenza A (H1N1) mutants and treated with UV-4B, oseltamivir, or vehicle. (**A**–**H**) BALB/c mice (n = 10/group) were infected i.n. (~1 LD_90_) with a wild-type recombinant virus or mutant recombinant viruses containing substitutions specific to UV-B treatment. Mice were treated via intragastric administration with UV-4B (100 mg/kg) or vehicle (water) three times daily or oseltamivir (20 mg/kg) twice daily for 5 days beginning 8 h post-challenge. **P < 0.01, ***P < 0.001 when compared to the vehicle group.

UV-4B treatment (100 mg/kg TID for 5 days beginning 8 h after infection) protected mice against lethal infection with all the recombinant wild-type and mutant IAV (p < 0.01 in all cases) (Fig. 5). Increased survival (60–100%) was observed with UV-4B treatment when mice were infected either with the wild-type or recombinant mutant viruses as compared to those treated with water alone (0–10%). Protection from weight loss was also apparent during the period of UV-4B treatment (day 4 p.i.) (Supplemental Fig. 3). Oseltamivir was included as a positive control compound and performed as expected against both recombinant wild-type virus and the recombinant virus with all three mutations (3 muts, M_PA_PB2) (Fig. 5A,H).

## Discussion

In this study, we evaluated the ability of mouse-adapted IAV (A/Texas/36/91 (H1N1)) to mutate *in vivo* in the presence of the host-targeted iminosugar UV-4B. Viruses passaged 5 times in mice in the presence or absence of UV-4B remained sensitive to UV-4B treatment in *in vivo* efficacy studies, providing evidence that UV-4B treatment does not promote the generation of viral escape mutants. Amongst the passaged viruses, relatively few minor variants were identified, and only three substitutions (one synonymous in PB2 and two nonsynonymous in M and PA) were specifically enriched in UV-4B-treated viruses at passage five. Recombinant viruses containing these mutations showed no detectable selective advantage and maintained their sensitivity to UV-4B treatment *in vivo*, corroborating a predicted high genetic barrier to escape mutations with host-targeted iminosugar antivirals^36^.

We identified minimal mutations in IAV after multiple passages in mice, and most of those mutations were not specifically associated with UV-4B treatment, indicating nonspecific selective pressure. Six of the 17 nonsynonymous substitutions identified were enriched in UV-4B-treated samples, which were limited to proteins that are not glycosylated by the host-cell machinery in the ER. Mice infected with recombinant viruses containing mutations in M, PA and/or PB2 were still susceptible to treatment with UV-4B; however, a slightly lower survival benefit (60–90%) was observed for some groups compared to the control group infected with the wild-type recombinant virus (100% survival). These differences could be due, in part, to challenges during the treatment procedure, possibly resulting in early deaths, or because UV-4B doesn’t consistently protect every animal from lethal infection, which would be more evident in a larger population. However, it is also plausible that these mutations could alter the HA/NA balance on the virions themselves or through some unknown mechanisms in the context of recombinant viruses. Although, it is important to note that all three of these unique substitutions were at sub-consensus levels in the passaged viruses, even after 5 rounds of passaging, suggesting limited to no selective advantage of these substitutions.

Based on the mechanism of action of UV-4B, it was expected that mutations unique to UV-4B treatment may be identified in viral glycoproteins, especially since this has been shown for selection of IAV in presence of mAbs^10,37^. The lack of variants in these proteins indicates that the virus did not acquire mutations necessary to overcome the selection pressure elicited by UV-4B, confirming a high genetic barrier for development of resistance. Unlike previous studies that showed concentration dependent positive selection of the IAV in the presence of oseltamivir and rapid emergence of oseltamivir resistant mutation after only one or two passages in mice^32^, our dN/dS analysis showed neutral selection during five successive passages in mice for four days in the presence of UV-4B, which accounts for a total of ~25–50 replication cycles of the virus (calculated based on ~5 to 10 replication cycles over the course of 4 days in mice/passaging^38^), further confirming the genetic barriers to escape UV-4B treatment. At the same time, as expected, passaging without drug showed negative selection (purifying selection) over time as this virus is already well adapted to the host (mice) and only purged changes that are deleterious on viral fitness in mice.

The generation of resistant mutants following UV-4B treatment was previously evaluated in mice following infection with DENV, a mosquito-borne pathogen that can cause severe and potentially life-threatening illnesses^27^. Like IAV, DENV requires the host’s ER α-glucosidase I and II enzymes for replication. Plummer *et al*., evaluated the evolution of two populations of passaged virus in the presence or absence of UV-4B in mice: DENV passaged in individual mice after a single passage, and pooled virus over the course of 4 serial passages. Similar to the results reported here for IAV, passaging of DENV in mice in the presence of UV-4B did not provide evidence for generation of viral escape mutants. Only 13 nonsynonymous SNPs in DENV were identified in pooled samples from at least one time point, and 12 of those 13 mutations were found in virus isolated from both the vehicle-treated and UV-4B-treated animals. However, the authors noted that by pooling samples for sequencing, some of the individual mouse-specific responses may have been diluted below detection. For this reason, combined with consideration of the normal passaging of DENV from invertebrate (mosquito) to vertebrate hosts rather than from vertebrate to vertebrate, they evaluated mutations acquired in DENV following a single passage in mice in the presence or absence of UV-4B. Synonymous and nonsynonymous mutations were identified in all DENV proteins after a single passage in mice. Unlike the current study, the dN/dS was higher when passaged in the presence of UV-4B compared to vehicle. Looking specifically at DENV glycosylated proteins (membrane, M; envelope, E; and NS1), nineteen nonsynonymous mutations were present at significant levels only in UV-4B-treated mice, possibly related to the mechanism of the drug. This was not observed for IAV in the present study, where no mutations specific to UV-4B treatment were identified in any of the viral glycoproteins.

Prior to this study, the development of drug resistant IAV strains was primarily evaluated for direct-acting antivirals. Unlike host-targeted iminosugars, approved anti-influenza virus drugs that are currently available directly target viral proteins (M2: amantadine and rimantadine; NA: oseltamivir, zanamivir, peramivir; PA: Xofluza^39^). The potential for influenza viruses to develop resistance to direct-acting antivirals is well established. Amantadine and rimantadine directly block the M2 proton channel to prevent the delivery of the viral genome into the cytoplasm of cells infected with IAV^40^. The Centers for Disease Control and Prevention (CDC) no longer recommends the use of amantadine or rimantadine to treat IAV infections in the United States because of the prevalence of resistant IAV strains that are no longer sensitive to treatment with these drugs^41^. Since the early 1970s, multiple groups have reported instances of resistance to amantadine and rimantadine^42–45^. As a result, neuraminidase inhibitors (NAI) are currently recommended by the CDC for treatment of influenza virus infections. NAIs, which inhibit the enzymatic activity of the viral NA protein, prevent virus release and spread and are effective against both IAV and IBV viruses. The active site of NA is highly conserved among all influenza viruses, making NA an ideal target for antiviral therapeutics directed against influenza virus^32,46^. However, mutations in any of the viruses that alter the shape/charge of the NA catalytic site can reduce binding and result in decreased sensitivity or resistance to treatment^47^ In addition, evidence shows that in the absence of NA-specific drugs, antibody escape in HA results in epistatic compensatory mutations in NA that could also result in drug resistance^1,48^.

Of the approved NAIs, oseltamivir is the only drug with sufficient bioavailability to be administered orally^49–55^. Oseltamivir-resistant strains can arise from a single mutation that results in a decrease in the binding affinity of oseltamivir to NA^56,57^. Additionally, combinations of multiple mutations can result in a synergistic or enhanced effect on oseltamivir antiviral resistance^32,58–61^ and can potentially restore mild reduced viral fitness that is observed with some single mutations^1,59^. As the potential risk for the transmission of drug resistant strains is a continuous concern, the impact on the efficacy of other antivirals against such resistant strains must be considered. Stavale *et al*., demonstrated that UV-4B maintains efficacy against an oseltamivir-resistant strain in a murine model of disease^25^. Here, we report that IAV passaged in mice five times in the presence of UV-4B did not acquire mutations that cause resistance to the commonly used anti-influenza drug oseltamivir. While the proposed mechanism for the antiviral activity of UV-4B is through perturbation of N-linked glycan processing, further studies need to be performed to determine which, if any, glycosylation sites on viral glycoproteins are affected and whether other mechanisms contribute to the anti-IAV activity of UV-4B. Using influenza virus reassortants, Hussain *et al*. demonstrated that changes in glycosylation of HA are the likely antiviral mechanism for iminosugars but more detailed analysis of which glycosylation sites are most sensitive has not yet been determined^62^.

It is interesting to note that UV-4B protects against fatal influenza infection *in vivo* (Fig. 2B,C); however, treatment with UV-4B does not robustly reduce viral replication in the lung (Fig. 2A) to a level that would be biologically significant. Based on these data, it appears that UV-4B may prevent influenza-induced morbidity and mortality through multiple biologic effects that may include mechanisms other than inhibition of viral replication. One such mechanism could be reduction in localized inflammation due to blunted cytokine responses. The freebase form of UV-4B (UV-4) itself does not induce a cytokine response in the blood of naïve, UV-4-treated mice^28^ or *in vitro* using mouse splenocytes, or human PBMC (Warfield *et al*., unpublished data) nor does it alter cytokine responses *in vitro* following mitogen stimulation (ex. PHA or LPS) (Warfield, *et al*., unpublished data). However, UV-4B treatment does result in reduced systemic cytokine responses in dengue virus infection mice^28^ and could also result in a reduced local (lung) cytokine responses following influenza infection. Alternately, other novel mechanisms that limit influenza pathogenesis by host-targeted antiviral iminosugars in addition to established mechanisms (i.e., direct impact on the hosts glycosylation pathway or indirectly by protein mis-folding due to ER stress) may be discovered^22^. The limited reduction in virus titer observed in the mouse lung following UV-4B treatment supports the potential use of UV-4B in a combination therapy. The effect of UV-4B treatment when co-administered with an NA inhibitor, and the potential for the development of drug resistant strains following combined treatment, should be considered and evaluated in future studies.

Taken together, our data suggests that the iminosugar UV-4B, which is effective against a broad range of influenza A and B viruses *in vitro* and *in vivo*, is at a low risk for selecting for mutant, drug-resistant influenza viruses. Iminosugars have positive drug-like properties and a history of safe use in humans, including five iminosugar compounds in clinical use: miglustat (Zavesca^®^) for the treatment of Gaucher’s disease and Niemann-Pick Type C; migalastat (Galafold™) for the treatment Fabry disease; and miglitol (Glyset^®^), acarbose (Precose^®^), and voglibose (Basen^®^) for the treatment or prevention of type II diabetes mellitus. Therefore, iminosugars remain a promising class of compounds for the future development of an antiviral therapeutic targeting a range of diverse viral pathogens, including DENV and influenza viruses.

## Materials and Methods

### Animal welfare, husbandry and observations

All procedures of the study were performed in accordance with the guidelines and protocols set and approved by the Noble Life Sciences or Utah State University Institutional Animal Care and Use Committee (IACUC). Both institutions are fully accredited by the Association for Assessment and Accreditation of Laboratory Animal Care International (AAALAC). Laboratory animals were observed and veterinary care was provided for all laboratory animals as required on a 24 hr basis, including weekends and holidays, in accordance with the Public Health Service Policy, U.S. Dept. of Agriculture (USDA) and AAALAC International requirements.

Female BALB/c mice (6–8 weeks of age) maintained under specific pathogen-free conditions were procured from Charles River Laboratories and were quarantined for >48 h prior to study initiation. During in-life study duration, all animals were observed carefully 2–3 times daily for changes in clinical signs, including morbidity and mortality. Survival and health were evaluated daily using a scoring system to assess signs of clinical disease (e.g. appearance of fur coat, posture, mobility, activity level and attitude) and provide a numerical value (1–7) directly related to disease severity as previously described^25^. Mice were euthanized when they met a 30% weight loss cut-off or scored at ≥6 in the standard scoring system. Animals were euthanized by administration of inhaled CO_2_ followed immediately by cervical dislocation and in accordance with the 2013 American Veterinary Medical Association (AVMA) Guidelines on Euthanasia.

### Passaging description

The original virus stock of the mouse-adapted influenza A/Texas/36/91 (H1N1) was obtained from the Baylor School of Medicine (kind gift of P. Wyde and B. Gilbert). Using 6-8-week-old female BALB/c mice, a new working virus stock (P0) was prepared using infected-lung homogenates and the LD_90_ was determined to be 52 PFU/mouse. Influenza A/Texas/36/91 (H1N1) was passaged successively five times in mice before determining the efficacy of UV-4B against the P5 virus. One group of 30 mice (P0) was challenged with influenza A/Texas/36/91 (H1N1) (~1 LD_90_ or ~52 PFU) intranasally and were treated via intragastric administration with either 100 mg/kg UV-4B or its vehicle (water) control starting one hour after infection. This treatment continued TID for seven days. A portion of these mice (n = 10) were observed daily for weight changes, morbidity (assessed using standard health scores) and mortality for 14 days. The remaining mice (n = 5) were sacrificed on Day 4 post-infection and their lungs were bisected. Half of each lung was immersed in RNALater for RNA extraction and subsequent virus sequencing. The other half of each lung was snap-frozen in liquid nitrogen and stored frozen until homogenization in PBS on wet ice. Homogenate samples from individual mice were maintained for titration with 0.2 mL from each mouse combined into a pool homogenate to be used in the next passage. Following virus titration of the lung homogenate pool, these viruses were then used as the challenge agent (P1-4) in the next round of virus challenge at ~52 PFU/mouse (~1 LD_90_) of input virus. Virus passaging continued for a total of five times.

### Lethal dose 90% (LD_90_) and efficacy determination of passage 5 viruses

The LD_90_ was determined for influenza viruses isolated from lungs after 5 passages in mice treated with UV-4B (P5A) or vehicle (P5B). Groups of ten BALB/c mice were used to test the lethality of six different challenge doses (1, 5, 10, 25, 50, or 100 PFU) of each virus following i.n. exposure. All mice were orally administered 100 µL of water TID starting 1 h p.i. for a total of ten days to mimic treatment. Mice were monitored daily for changes in health (assessed using standard scoring system), weight, and mortality for 14 days.

Groups of 30 mice were challenged with either P5 virus isolated from animals treated with UV-4B (P5A, ~1 LD_90_ = 25 PFU) or from animals treated with the vehicle control (P5B, ~1 LD_90_ = 10 PFU). One hour following infection, the animals in these groups were treated via intragastric administration with either 100 mg/kg of UV-4B (N = 10), vehicle control (N = 10), or 20 mg/kg of the positive control, oseltamivir. The treatments with UV-4B and vehicle continued TID for seven days, while the treatment with oseltamivir continued twice a day for five days. Mice were observed daily for 14 days for changes in health, weight, and mortality.

### Influenza virus next-generation sequencing

Viral RNA from the infected lung lysates were individually evaluated using multi-segment reverse transcriptase-polymerase chain reaction (M-RT-PCR)^63^, followed by library preparation using the Nextera DNA library preparation kit (Illumina) and the Ion Xpress™ Plus Fragment Library Kit (Thermo Fisher Scientific) for sequencing using Illumina MiSeq and Ion Torrent PGM, respectively, to overcome platform specific errors. The IAV genomic RNA segments were simultaneously amplified from 3 µl of purified RNA using M-RT-PCR^34^. Illumina libraries were prepared from these M-RT-PCR products using the Nextera DNA Sample Preparation Kit (Illumina, Inc., San Diego, CA, USA) with half-reaction volumes. PCR products were quantified using QIAxcel (Qiagen, Hilden, Germany), and 25 ng of DNA amplicons for each sample were tagmented (fragmented and tagged) at 55 °C for 5 min. Tagmented DNA amplicons were cleaned with the ZR-96 DNA Clean & Concentrator Kit (Zymo Research Corporation, Irvine, CA, USA) and eluted in 25 µl resuspension buffer. Illumina sequencing adapters and barcodes were added to tagmented DNA via PCR amplification by combining 20 µl tagmented DNA with 7.5 ul Nextera PCR Master Mix, 2.5 ul Nextera PCR Primer Cocktail, and 2.5 µl of each index primer (Integrated DNA Technologies, Coralville, IA, USA) for a total volume of 35 µl per reaction. Five cycles of PCR were performed as per the Nextera DNA Sample Preparation Kit protocol (3 min at 72 °C, denaturation for 10 sec at 98 °C, annealing for 30 sec at 63 °C, and extension for 3 min at 72 °C) to create a dual-indexed library for each sample. After PCR amplification, 10 µl of each library derived from M-RT-PCR products were pooled into a 1.5-ml tube; separately, 10 µl of each library derived from HA-specific amplicons were pooled into a 1.5-ml tube. Each pool was cleaned two times with Ampure XP Reagent (Beckman Coulter, Inc., Brea, CA, USA) to remove all leftover primers and small DNA fragments. The first and second cleanings used 1.2x and 0.6x volumes of Ampure XP Reagent, respectively. The cleaned pool derived from M-RTPCR products was sequenced on the Illumina HiSeq 2000 instrument (Illumina, Inc.) with 100-bp paired-end reads, while the cleaned pool derived from HA-specific amplicons was sequenced on the Illumina MiSeq v2 instrument with 300-bp paired-end reads.

Separately, influenza M-RT-PCR products were randomly amplified and prepared for NGS using a sequence-independent single-primer amplification (SISPA) method as previously described^64^. The methodology used 100 ng of amplified viral DNA that was denatured in the presence of DMSO and a chimeric oligonucleotide containing a known 22-nt barcode sequence followed by a 3′ random hexamer. A Klenow reaction was prepared with the denatured DNA template by adding NEB buffer II, 3′-5′ exo- Klenow (New England Biolabs, Ipswich, MA, USA), and dNTPs (Thermo Fisher Scientific, Waltham, MA, USA). The Klenow reaction was incubated at 37 °C for 60 min, followed by incubation at 75 °C for 10 min. The resulting cDNA was randomly amplified by PCR using the Promega GoTaq Hot Start Polymerase (Promega Corporation, Madison, WI, USA) for 35 cycles (denaturation: 30 sec, 94 °C; annealing: 30 sec, 55 °C; extension: 48 sec, 68 °C). PCR reactions contained primers corresponding to the known 22-nt barcode sequence from the oligonucleotide utilized in the previous Klenow step. The resulting cDNA was then treated with Exonuclease I at 37 °C for 60 min, followed by incubation at 72 °C for 15 min. SISPA products were normalized and pooled into a single reaction that was purified using the QIAquick PCR purification kit (Qiagen). Pooled samples were further purified to select for SISPA products 300–500 bp in size for Illumina Miseq paired-end (2 × 300) sequencing.

For additional sequencing coverage, samples were re-sequenced using the Ion Torrent platform. M-RT-PCR products were sheared for 7 min, and Ion-Torrent-compatible barcoded adapters were ligated to the sheared DNA using the Ion Xpress Plus Fragment Library Kit (Thermo Fisher Scientific) to create 400-bp libraries. Libraries were pooled in equal volumes and cleaned with the Ampure XP Reagent. Quantitative PCR was performed on the pooled, barcoded libraries to assess the quality of the pool and to determine the template dilution factor for emulsion PCR. The pool was diluted appropriately and amplified on Ion Sphere Particles (ISPs) during emulsion PCR on the Ion One Touch 2 instrument (Thermo Fisher Scientific). The emulsion was broken, and the pool was cleaned and enriched for template-positive ISPs on the Ion One Touch ES instrument (Thermo Fisher Scientific). Sequencing was performed on the Ion Torrent PGM using a 318v2 chip (Thermo Fisher Scientific).

### Virus genome assembly and variant analysis

For virus sequence assembly, all sequence reads were sorted by barcode, trimmed, and *de novo* assembled using CLC Bio’s *clc_novo_assemble* program (Qiagen). The resulting contigs were searched against custom full-length influenza segment nucleotide databases to find the closest reference sequence for each segment. All sequence reads were then mapped to the selected reference influenza A virus segments using CLC Bio’s *clc_ref_assemble_long* program.

Minor allele variants (i.e., nucleotide sequences that differ from the reference sequence and are supported by sequencing reads at or above a pre-defined threshold) were identified using *FindStatisticallySignificantVariants* (*FSSV*) software (http://sourceforge.net/projects/elvira/). The *FSSV* software applied statistical tests to minimize false-positive Single Nucleotide Polymorphism (SNP) calls generated by Illumina sequence-specific errors (SSEs)^65^. SSEs usually result in false SNP calls if sequences are read in one sequencing direction. The *FSSV* analysis tool requires observing the same SNP at a statistically significant level in both sequencing directions. Once a minimum minor allele frequency threshold and significance level are established, the number of minor allele observations and major allele observations in each direction and the minimum minor allele frequency threshold are used to calculate p-values based on the binomial distribution cumulative probability. If the p-values calculated in both sequencing directions are less than the Bonferroni-corrected significance level, then the SNP calls are accepted. A significance level of 0.05 (Bonferroni-corrected for tests in each direction to 0.025) and a minimum minor allele frequency threshold of 3% were applied for this analysis, and the consensus sequence from the input parental virus was used as the reference sequence for each sample.

Differences in the consensus sequence compared to the reference sequence were identified using CLC Bio’s *find_variations* software. The identified consensus and minor allele variations were analyzed by assessing the functional impact on coding sequences or other regions based on overlap with identified features of the genome. For each sample, the reference sequence was annotated using *VIGOR* software^31^, and then the variant data and genome annotation were combined using *VariantClassifier* software^66^ to produce records describing the impacts of the identified variations.

### Recombinant viruses

To generate the recombinant A/Texas/36/1991 (H1N1) viruses, the appropriate gene segments were cloned into reverse genetics plasmids as previously described^33^. Substitutions in PA, PB and M were performed using site-directed mutagenesis. Wild-type and recombinant mutant viruses A/Texas/36/1991 (H1N1) were rescued using reverse genetics systems with 293 T and MDCK co-culture as previously described^35,63,67^.

### Virus titration

The 50% tissue culture infectious dose (TCID_50_) assay was performed for *in vitro* titrations of IAV. Briefly, the supernatants from the lung homogenates were evaluated using 10-fold serial dilutions in 96-well plates (six dilutions total, 10^−2^ to 10^−7^) to determine the viral titers. MDCK cells were seeded at a density of 1 × 10^4^ cells per well in 100 µL of medium into 96-well plates and incubated overnight (20–24 h). Cells were infected with the dilutions in 9 replicates, three plates per yield dilution, with one column serving as the negative control. Cells were incubated at 35 °C until the cytopathic effect (CPE) in wells containing infected cells remained constant (6–10 days). The cells were then fixed with 5% glutaraldehyde and stained with 0.1% crystal violet to measure CPE. The number of infected wells at each dilution was used to calculate the TCID_50_ value based on the Reed-Muench method^68^. The calculation of PFU from TCID_50_ is based on the ratio PFU/TCID_50_ of the limiting dilution which would infect 50% of the challenged cell layers^69^.

### LD_90_ and efficacy determinations of recombinant IAV

To determine the LD_90_ of each recombinant virus (wild-type or substituted), groups of five mice were exposed intranasally (75 µl) to an individual virus using dilutions of 1:100, 1:320, 1:1000, and 1:3200. A group of 5 mice were not infected and served as weight/survival controls. Mice in all groups were treated with water via intragastric administration 3 times daily for 7 days, beginning 8 hours after infection. Mice were monitored for weight loss, morbidity and mortality as described above. LD_90_ values were graphically estimated and used to set challenge inoculum dilutions for each individual recombinant virus (ranging from 1:320-1:1000).

Groups of mice were exposed intranasally (75 µl) to the wild-type or recombinant viruses at ~1LD_90_. Groups of mice were treated with UV-4B (100 mg/kg/dose or vehicle (water) via intragastric administration TID for 5 days beginning 8 hours after infection. For two of the viruses, a group treated with oseltamivir was also included as a positive control, where mice were treated with oseltamivir (10 mg/kg/day) twice daily for 5 days beginning four hours after infection. Mice were monitored for weight loss, morbidity and mortality as described above.

### Statistical analyses

Statistical analyses were performed using GraphPad Prism. Virologic titers were log_10_‐transformed and statistical analysis between vehicle- and UV‐4B‐treated groups at each passage performed using an unpaired parametric t‐test. Survival analysis was performed using the Kaplan-Meier graphing method and log-rank test. Linear regression analysis was performed to test if slopes of the number of substitutions or the dN/dS ratio over passage number were significantly different than zero.

### Accession number of sequences

All the sequences are submitted to GenBank with access no MK615188-MK615595.

## Supplementary information

Dataset 1
