# Supplementary material for: Lack of selective resistance of influenza A virus in presence of host-targeted antiviral, UV-4B
Source: Sci Rep. 2019 May 16;9:7484. doi: 10.1038/s41598-019-43030-y (PMC6522537; doi:10.1038/s41598-019-43030-y)

Lack of selective resistance of influenza A virus in presence of host-targeted antiviral, UV-4B.

Kelly L. Warfield[1#], Kaitlyn Schaaf[2], Lisa Evans DeWald[1], Kevin Spurgers[1], Wei Wang[3, a], Eric Stavale[4, b], Michelle Mendenhall[5], Meghan H. Shilts[2, 3], Timothy B. Stockwell[3, c], Dale L. Barnard[5], Urban Ramstedt[6, b], Suman R. Das[2, 3,#]

[1] Emergent BioSolutions, Gaithersburg, MD 20879, USA.

[2] Department of Infectious Diseases, Vanderbilt University Medical Center, Nashville, TN 37232, USA

[3] Infectious Diseases Group, J. Craig Venter Institute, Rockville, MD 20852, USA

[4] Integrated Biotherapeutics Inc., Gaithersburg, MD 20878, USA.

[5] Institute for Antiviral Research, Utah State University, Logan, UT 84322-5600, USA

[6] Unither Virology, LLC, Silver Spring, MD 20910, USA

[*] **Current affiliations:**

[a] Cellular Biology and Viral Immunology Section, DIR, National Institute of Health, Bethesda, MD, 20892, USA

[b] Abviro, 4800 Hampden Lane, Bethesda, MD 20814, USA

[c] National Biodefense Analysis and Countermeasures Center, 8300 Research Plaza, Fort Detrick, MD 21702, USA

[#] **Corresponding authors:**

Suman R Das: suman.r.das@vanderbilt.edu

Kelly L. Warfield: warfieldk@ebsi.com

Running Title: High genetic barrier to escape UV-4B

Key Words: Influenza virus, iminosugar, glycosylation, antiviral, UV-4B

Journal: Scientific Reports

SUPPLIMENTARY FIGURE LEGENDS

Supplemental Figure 1: Relative average weight of mice infected with decreasing challenge doses of wild-type or mutant recombinant viruses. BALB/c mice (n=10/group) were challenged i.n. with varying dilutions of wild-type or mutant recombinant viruses and the group weight was measured daily through the end of the study (Day 14). The percent average weight loss was calculated for each group relative to Day 0 weights.

Supplemental Figure 2: Survival outcome of mice infected with decreasing challenge doses of wild-type or mutant recombinant viruses. BALB/c mice (n=10/group) were challenged i.n. with varying dilutions of wild-type or mutant recombinant viruses. Survival was monitored through the end of the study (Day 14) to determine the LD_{90} of each virus.

Supplemental Figure 3. Relative average weight of mice infected with recombinant wild-type or mutant viruses. BALB/c mice (n=10/group) were infected with various recombinant influenza viruses and orally treated with UV-4B (100mg/kg) or vehicle (water) three times daily or oseltamivir (20 mg/kg) twice daily for 5 days beginning 8 h post-challenge. Average group weights were determined daily through the end of the study (Day 14).

SUPPLEMENTAL TABLES

Supplemental table 1: Summary of survival results for P5 efficacy test.

**Treatment**	**Challenge virus**	**Percent Survival**	**Median Survival (Days)[a]**	**P-value** (significance between UV-4B-treated and vehicle/oseltamivir-treated groups)
UV-4B	P5A (UV-4B passaged virus)	100	Undefined	N/A
Vehicle		0	13	<0.0001
Oseltamivir		100	Undefined	1.00
UV-4B	P5B (Vehicle passaged virus)	100	Undefined	N/A
Vehicle		0	9	<0.0001
Oseltamivir		100	Undefined	1.00

[a] If survival did not exceed 50% at the longest time point then the median survival cannot be computed. Thus, it is ‘undefined’.

Supplemental Table 2: Comparison of the mouse-adapted influenza A/Texas/36/91 (maH1N1) virus challenge stock with GenBank sequences.

Segment Number	Protein	No. of NT differences	CDS location (NT)	CDS location (AA)	Sequence Difference Comparison[a,b]	Difference (NT)	Difference (AA)
1	PB2	1	792	264	NCBI2 vs NCBI1/maH1N1	AG**A** vs AG**G**	synonymous
2	PB1	2	1636	546	NCBI1/NCBI2 vs maH1N1	**A**TG vs **C**TG	M vs L
			2073	691	NCBI1/NCBI2 vs maH1N1	G**G**T vs G**A**T	synonymous
3	PA	1	1343	448	NCBI1/NCBI2 vs maH1N1	G**C**A vs G**A**A	A vs E
4	HA	7	310	104	NCBI1/NCBI2 vs maH1N1	**A**AT vs **G**AT	N vs D[c]
			360	120	NCBI1/NCBI2 vs maH1N1	GA**G** vs GA**T**	E vs D
			472	158	NCBI1 vs NCBI2/maH1N1	**A**AA vs **G**AA	K vs E[d]
			508	170	NCBI1 vs NCBI2/maH1N1	**G**AG vs **A**AG	E vs K[e]
			560	187	NCBI1/NCBI2 vs maH1N1	G**A**G vs G**G**G	E vs G
			607-608	203	NCBI1 vs NCBI2 vs maH1N1	**AG**G vs **GG**G vs **GA**G	R vs G vs E
5	NP	4	59	20	NCBI1 vs NCBI2/maH1N1	C**C**G vs C**A**G	P vs Q
			293	98	NCBI1/NCBI2 vs maH1N1	A**A**G vs A**G**G	K vs R
			453	151	NCBI2 vs NCBI1/maH1N1	AC**A** vs AC**T**	synonymous
			838	280	NCBI1 vs NCBI2/maH1N1	**G**TG vs **A**TG	V vs M
6	NA	1	1289	430	NCBI1/NCBI2 vs maH1N1	C**G**A vs C**A**A	R vs Q
7	M1	0					
	M2	2	131	44	NCBI1/NCBI2 vs maH1N1	G**A**T vs G**G**T	D vs G
			288	96	NCBI2 vs NCBI1/maH1N1	CT**G** vs CT**A**	synonymous
8	NS1	1	307	103	NCBI1/NCBI2 vs maH1N1	**T**TC vs **G**TC	F vs V
	NEP/NS2	1	348	116	NCBI1/NCBI2 vs maH1N1	TT**T** vs TT**C**	synonymous

AA – Amino Acid; NT – Nucleotide

[a] NCBI1 = sequence submitted by JCVI, accessions CY033600 to CY033605, CY033655, CY033656

[b] NCBI2 = sequence submitted by Baylor, accessions DQ508886 to DQ508893

[c] Removes a potential glycosylation site (**N**GT to **D**GT) [d] Immediately before a Ser; may result in a change

Supplemental Figure 1

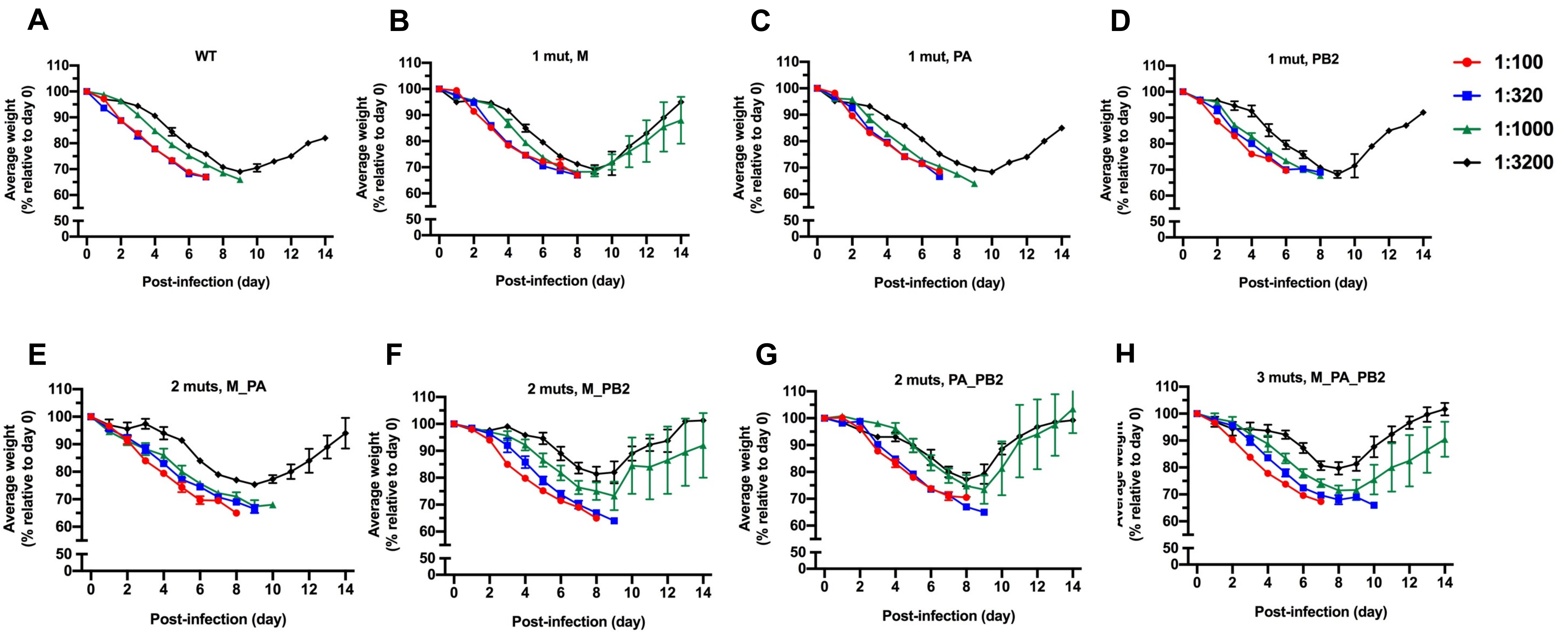

Supplemental Figure 2

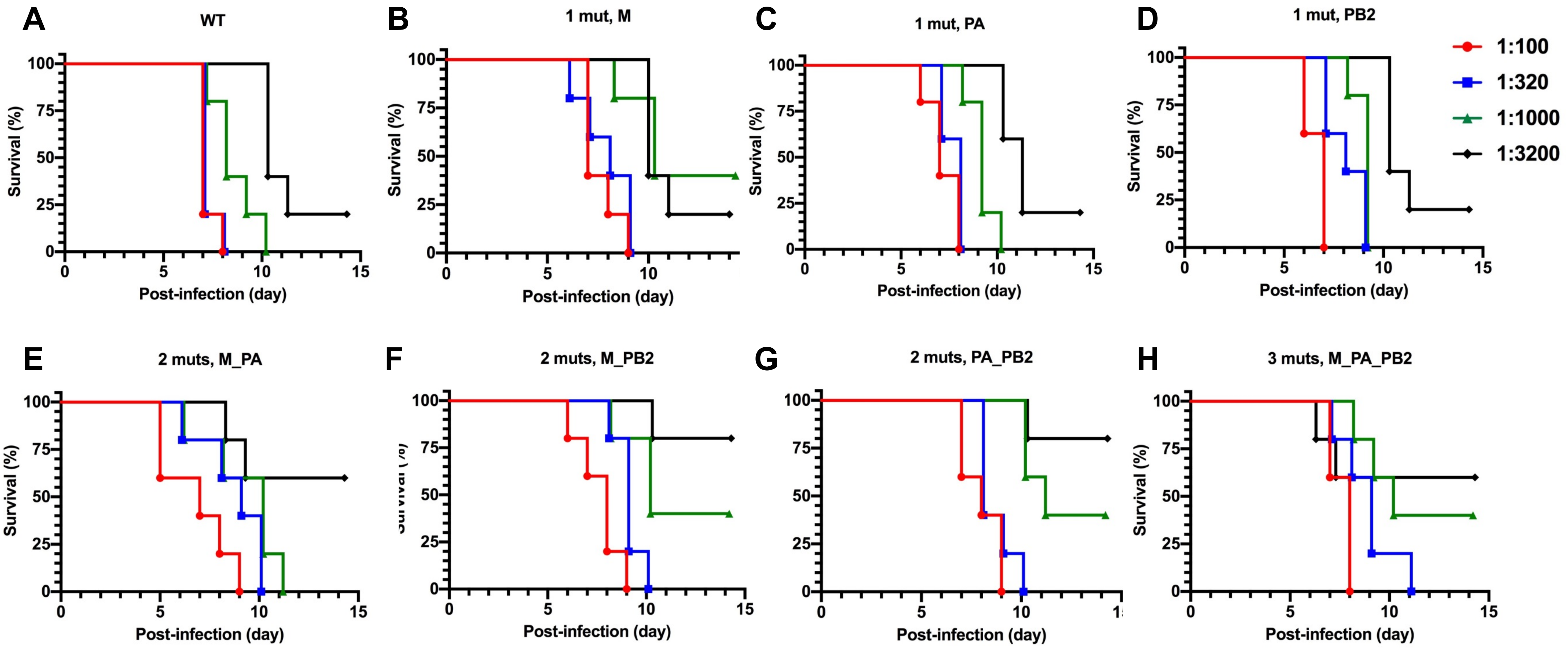

Supplemental Figure 3

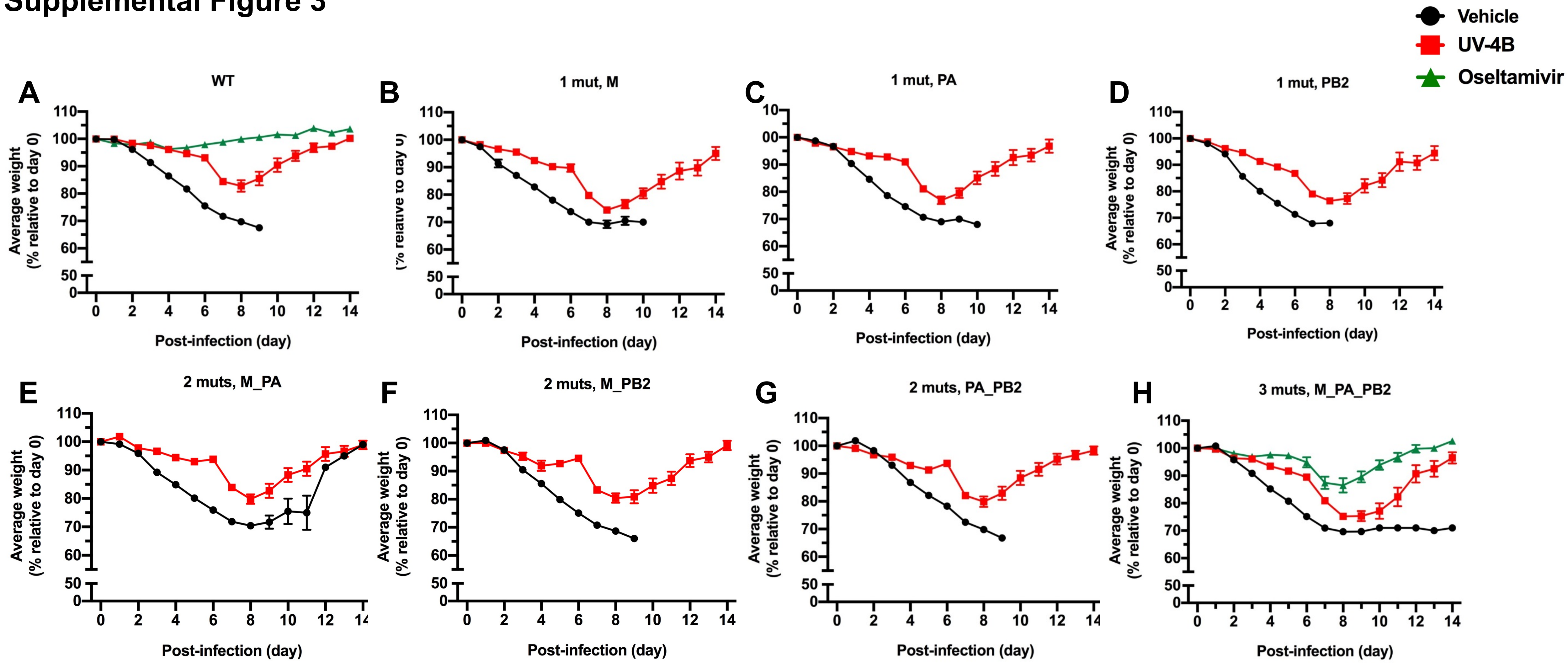

Supplement: Supplementary file 1 — Dataset 1 [file 41598_2019_43030_MOESM1_ESM.pdf]
